# Which is more important for classifying microbial communities: who's there or what they can do?

**DOI:** 10.1038/ismej.2014.157

**Published:** 2014-08-29

**Authors:** Zhenjiang Xu, Daniel Malmer, Morgan G I Langille, Samuel F Way, Rob Knight

**Affiliations:** 1 Biofrontiers Institute, University of Colorado, Boulder, CO, USA; 2 Department of Pharmacology, Dalhousie University, Halifax, NS, Canada; 3 Department of Computer Science, University of Colorado, Boulder, CO, USA; 4 Howard Hughes Medical Institute, University of Colorado, Boulder, CO, USA

Classification is a machine-learning approach to develop predictive models that can classify samples into categories correctly. In microbial studies, these categories include disease states and habitats. An ongoing question in microbial ecology is the correct level of analysis to use in order to best discriminate biologically relevant samples. Many studies use the *16S rRNA* gene as a taxonomic marker, and then ask how effectively the taxonomic profiles obtained from this marker classify or cluster different microbial communities according to their sample types. Interestingly, the answer may depend on the question being asked. For phylogenetic analysis, different levels of resolution in grouping are differentially successful at different classification tasks. These classification tasks include separating different samples by the person they came from (which depends on fine distinctions among very closely related strains or species), and separating lean from obese individuals (where very broad groups of taxa are more effective) (Knights *et al.*, 2011b).

A related controversy is whether taxonomy is the right level of analysis at all: might we not instead expect that function would be substantially more important for classifying biologically meaningful groups of samples than who is providing those functions? For example, a pair of grasslands is immediately distinguishable from a pair of forests just by looking at them. This is true even if the plants that make up the relevant grasslands and the relevant forests are not closely related to one another phylogenetically. We might expect the same to be true in the microbial world. Therefore, we might expect that classifying the members of a microbial community in terms of molecular function would provide far more discriminatory power than looking at taxonomic profiles, especially because taxonomic profiles are extremely variable in cases where functional profiles are more stable (Turnbaugh *et al.*, 2008; Human Microbiome Project Consortium, 2012). So there is likely to be less noise in interpreting functional profiles; on the other hand, functional profiles contain less variation, so perhaps there is less covariation with clinically or environmentally important parameters to explain.

Recently, the development of methods to predict functional profiles from taxonomic profiles of the same data, PICRUSt (Langille *et al.*, 2013), allows us to address this question. PICRUSt is a tool that predicts the gene content of a microbial community from a marker gene survey, using an existing database of microbial genomes. Knights *et al.* used published data sets, including Costello *et al.* Body Habitats (CBH), Costello *et al.* Skin Sites (CSS), Costello *et al.* Subject (CS), Fierer *et al.* Subject (FS), and Fierer *et al.* Subject-Hand (FSH), to ask how effectively we can accurately classify samples from different body sites, different individuals, and different clinical states (Knights *et al.*, 2011a). Using the same data sets, we asked whether functional profiles as predicted by PICRUSt provided better or worse ability to classify samples according to biologically meaningful categories with the Random Forest classifier. Random Forest is an ensemble classification method that fits a set of decision trees on subsamples of the data set, and then combines the results to improve classification accuracy. The key input features (operational taxonomic units (OTUs) or genes in this case) can be ranked by their contributions in distinguishing samples from different categories (Supplementary Figures S1 and S2 and Supplementary Tables S1 and S2) (Liaw and Wiener, 2002; Kuhn, 2008).

The results were intriguing (Figure 1): functional classification performed better in one task, CBH (the easiest of the classification tasks), worse in three, CS, FS and FSH, and not significantly different in the last one, CSS. Noticeably, for both the challenging classification tasks with poor accuracies, CSS and FSH, where the differences in taxonomic composition between classes are subtle, the PICRUSt-predicted functional profile does not offer any improvement upon microbial composition data alone.

The observation that adding functional information does not improve classification accuracy is surprising. However, one possible reason for the lack of improvement relative to taxonomy that we needed to control for is that the functional predictions might be of insufficient quality. To test this hypothesis, we used Human Microbiome Project (HMP) data set from the PICRUSt paper, where paired shotgun metagenomic annotation and 16S rRNA profile data from the same samples were available. Presumably, the functional profile from metagenomic annotation is better to functionally characterize a microbial community than that inferred with PICRUSt. For this data set, the classification based on PICRUSt-predicted functions is actually slightly better than those based on 16S profile and metagenomic annotation, although there is no significant distinction between the latter two (Figure 1). Consequently, we can conclude that, for environments with enough reference genomes already in the database, PICRUSt provides information as good for classification as the direct functional assignment of the shotgun reads, although in either case functional information does not seem to improve classifier accuracy.

The results have several important implications for ecological studies of complex microbial communities. First, shotgun metagenomic and other functional studies are still far more expensive than 16S rRNA profiling, but might actually provide worse results if the goal is to obtain biomarkers for specific physiological or ecological states. Second, for multi-omics studies, different levels of function probably need to be examined empirically to understand which provides the best biomarkers—the study we performed here on the HMP data, examining 16S rRNA versus shotgun data, should be repeated at all multi-omics levels as they are acquired, for example, in HMP2. Finally, the underlying functional databases, which at present provide only relatively coarse-grained functional assignments, may need to be improved substantially before we are able to use functional genes for environmental classification in the way that taxonomic markers are already successful, particularly for environments that are underrepresented in the databases. Of course, there are other reasons for doing shotgun metagenomics, ranging from assembly of novel genomes to strain-level tracking of microbes over time, and functional assignments either from shotgun metagenomics or PICRUSt predictions can be immensely valuable for gaining functional insight into a given set of samples. However, improving our ability to classify samples into biologically meaningful categories is apparently not among the reasons to pursue functional, as opposed to taxonomic, characterization of microbial communities. Nevertheless, new technologies and better bioinformatic tools, such as longer sequencing reads, better annotation databases, tools to better predict gene and operon structures, and tools that interrogate single-nucleotide polymorphism-level data will be essential for providing more detailed and accurate functional annotations. These annotations will distinguish even more subtle differences between microbial samples, and help us understand the microbial world.

## Figures and Tables

**Figure 1:**
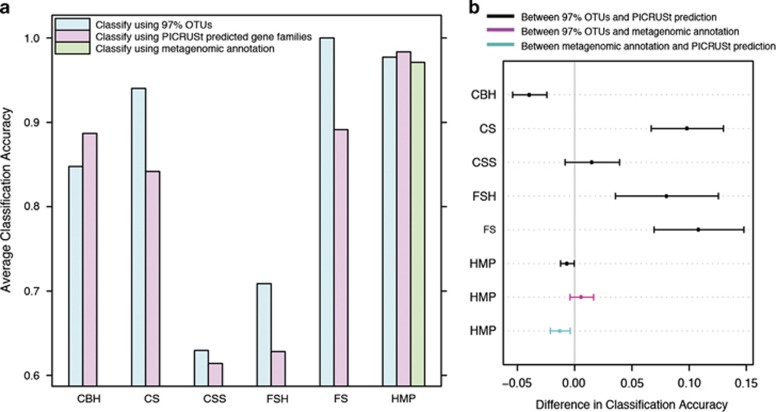
Accuracy of supervised classification. Random Forest classification model was performed, using caret (Kuhn, 2008) R package, with 5 repeats of 10-fold cross validation on CBH, CS, CSS, FSH, FS (from Knights *et al.*, 2011a and following the same naming) and HMP (from Langille *et al.*, 2013; other data sets with paired shotgun and 16S sequences are too small in sample size for classification) data sets. Kappa statistic is used here as measure of accuracy to assess the agreement between predicted classification and reality. (**a**) The average accuracies using as input the OTUs clustered at 97% sequence similarity, the functional profile predicted from the OTUs using PICRUSt or the annotation from shotgun metagenomic sequences (for HMP data set only). (**b**) The pairwise comparisons between the accuracies using those three inputs as predictive features. The error bars indicate 95% confidence interval.
