# Supplementary material for: Which is more important for classifying microbial communities: who's there or what they can do?
Source: ISME J. 2014 Aug 29;8(12):2357–9. doi: 10.1038/ismej.2014.157 (PMC4260698; doi:10.1038/ismej.2014.157)
Supplement: Supplementary Information [file ismej2014157x1.doc]

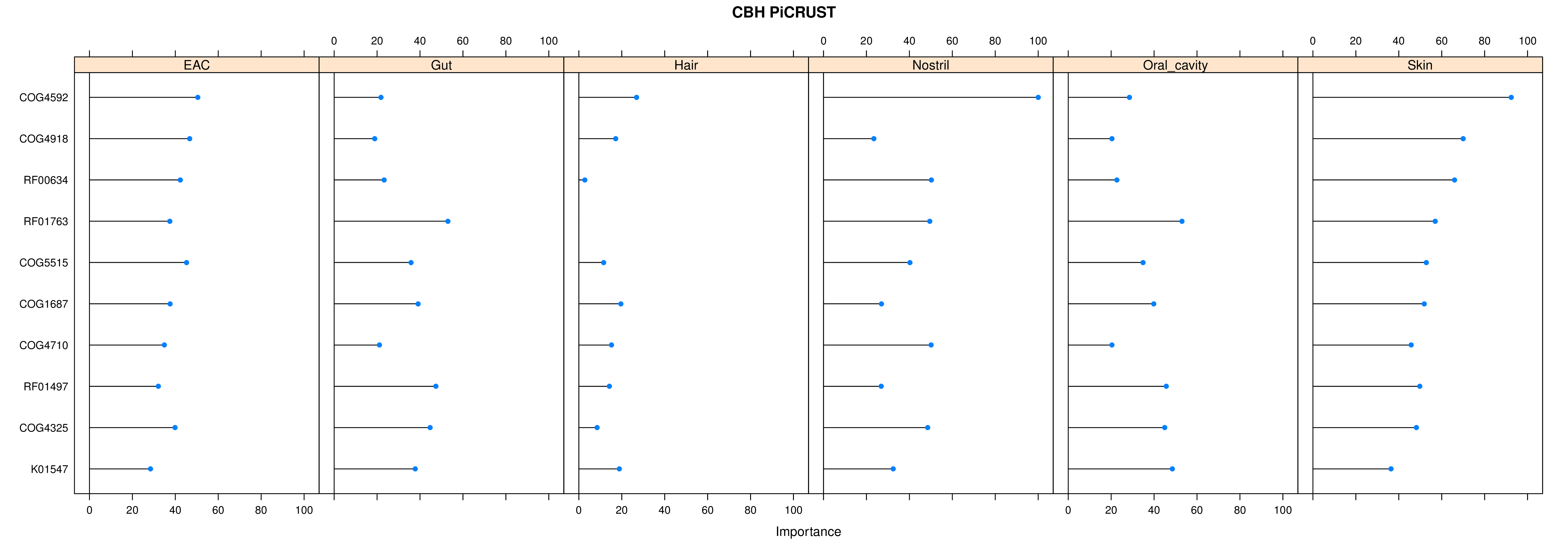

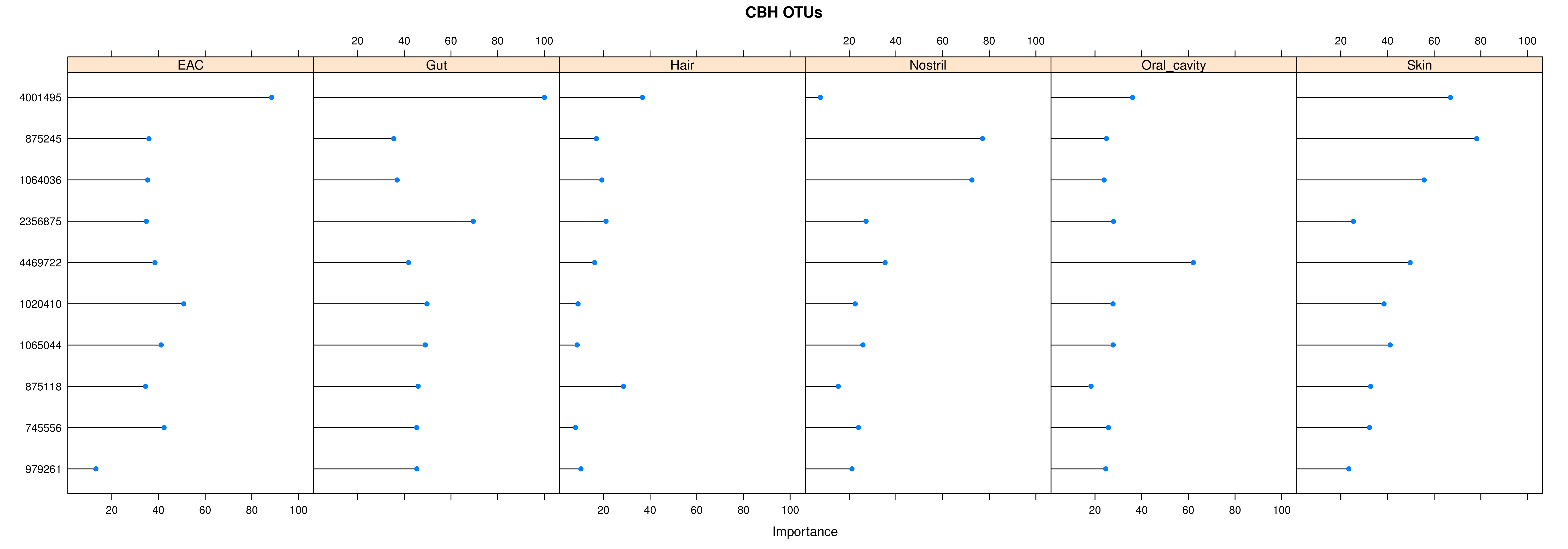


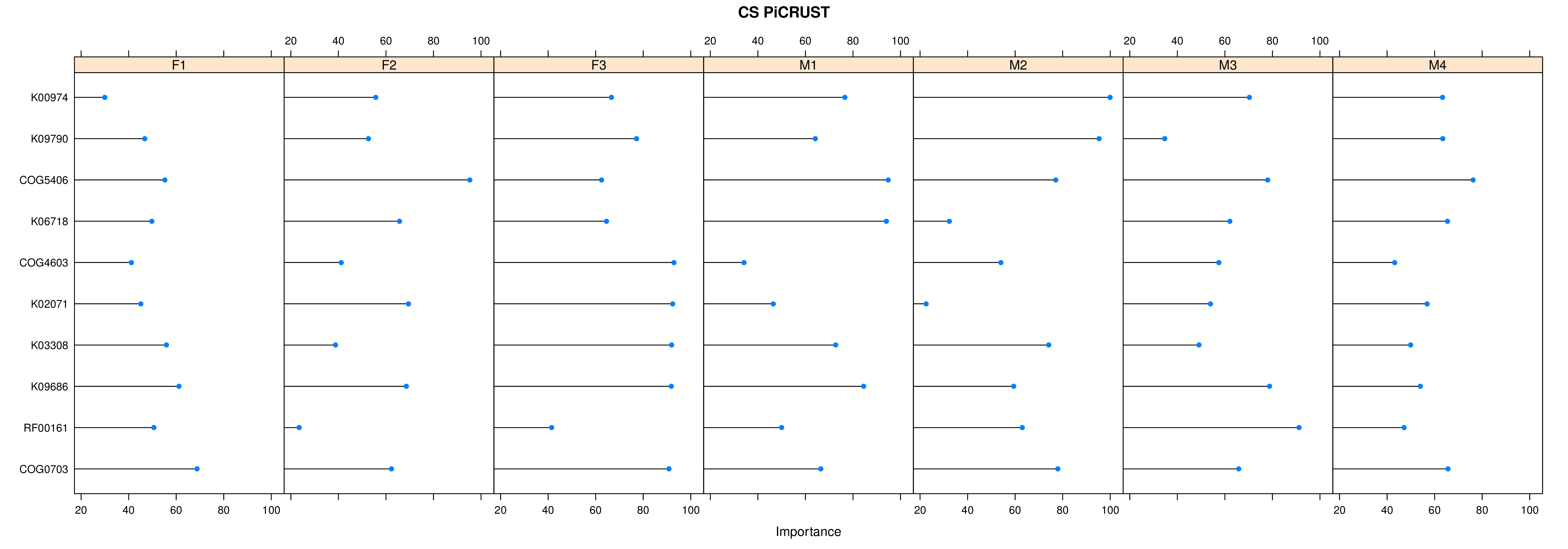

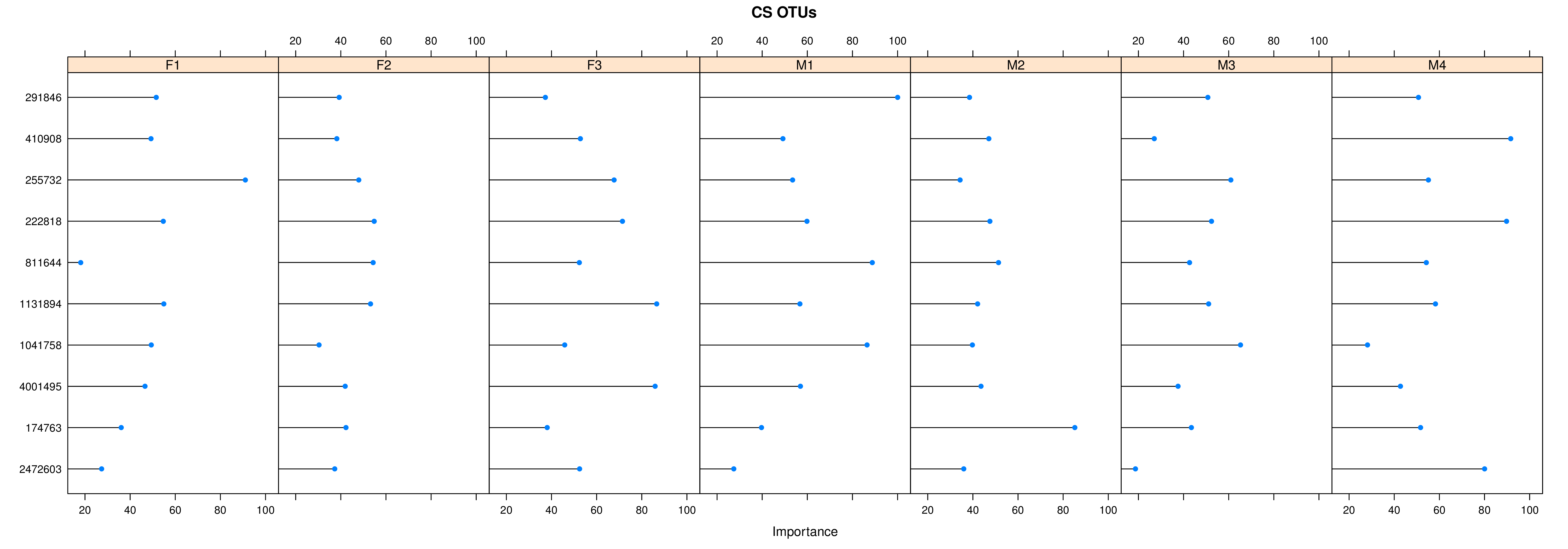


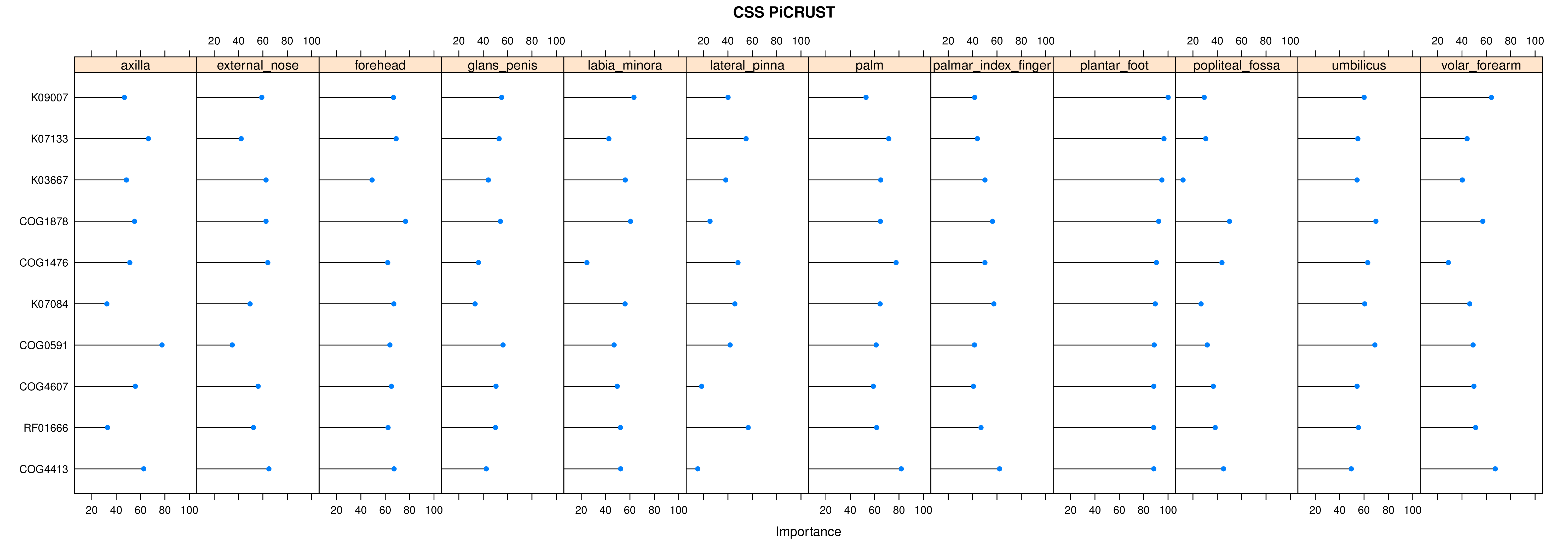


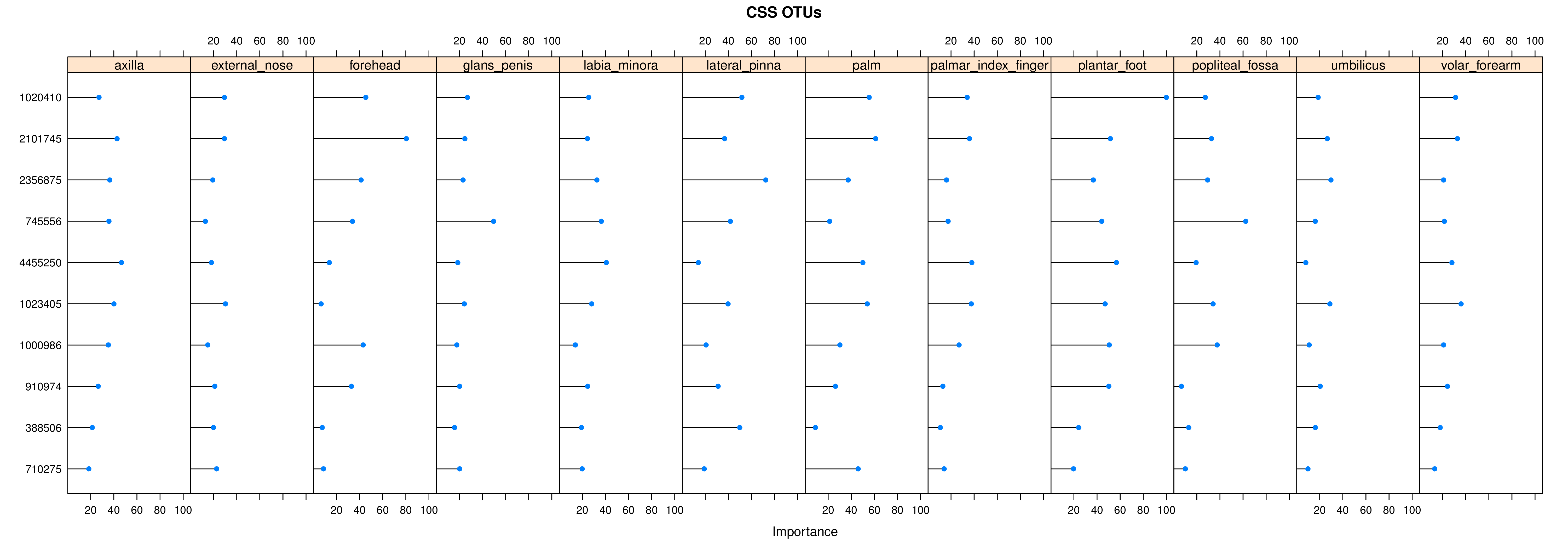


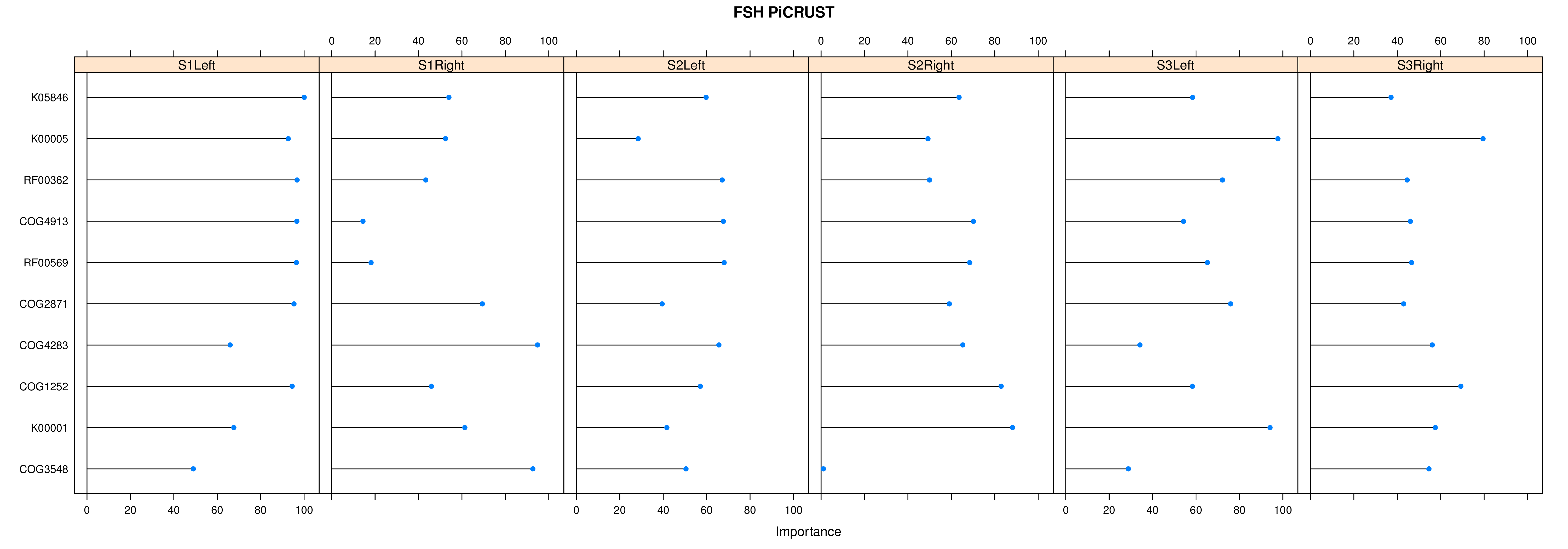

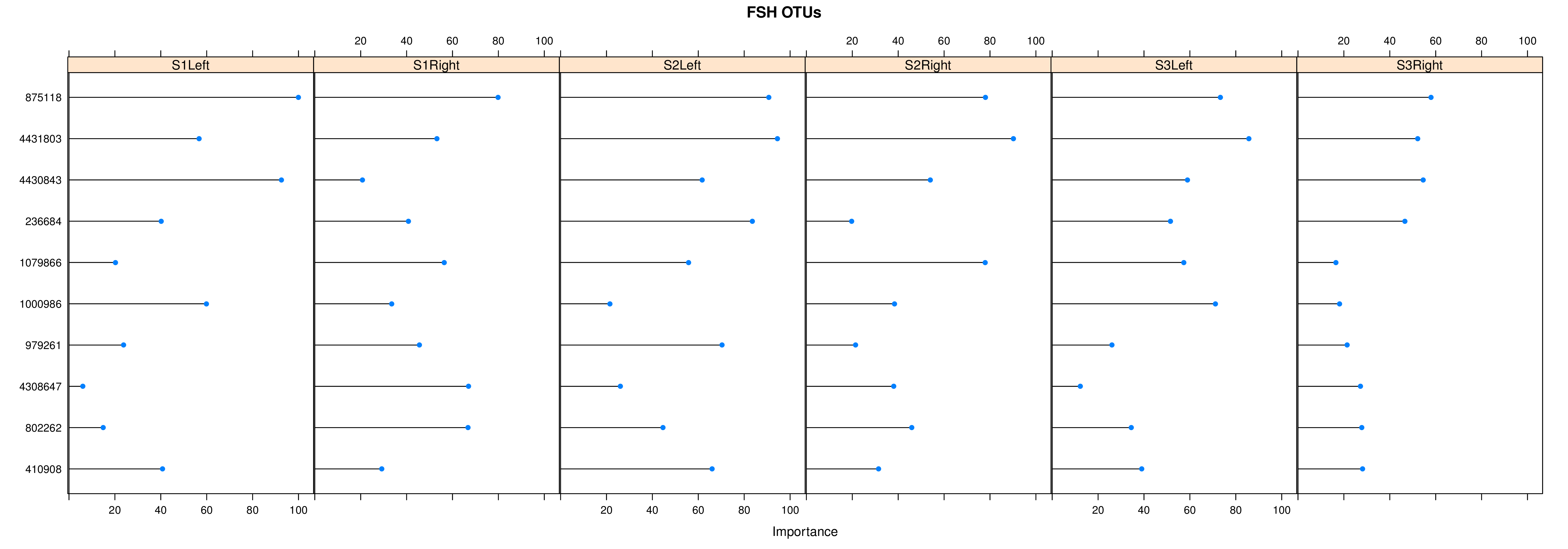

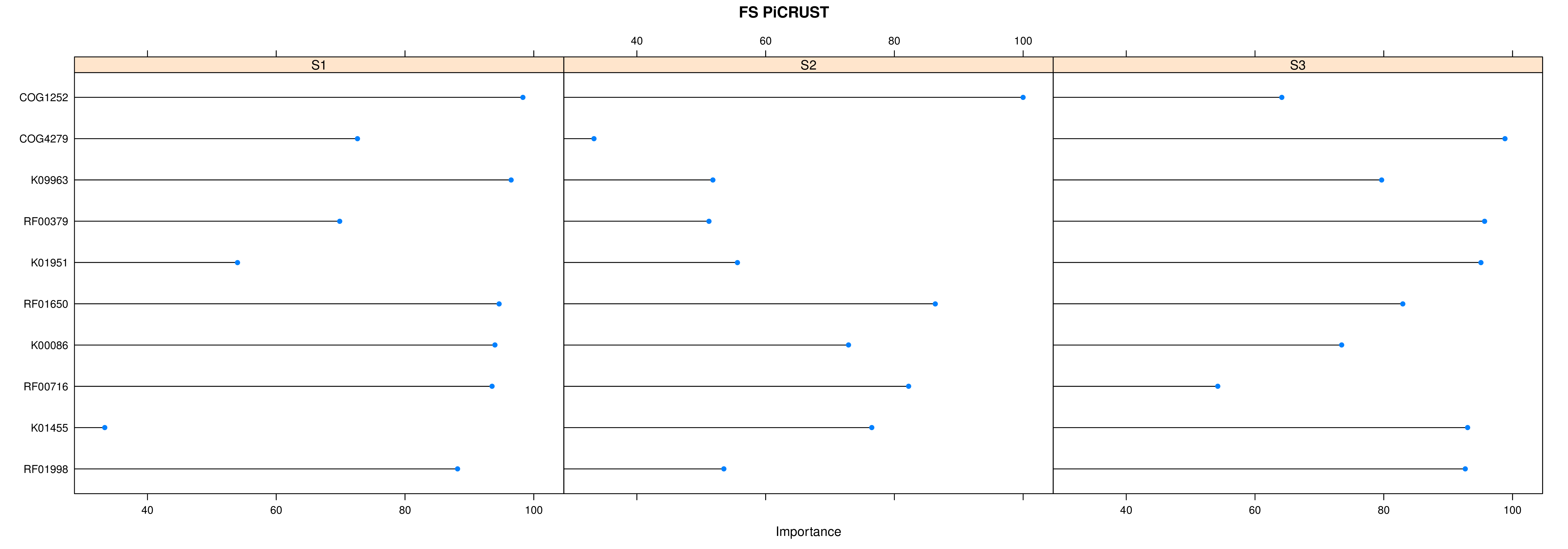

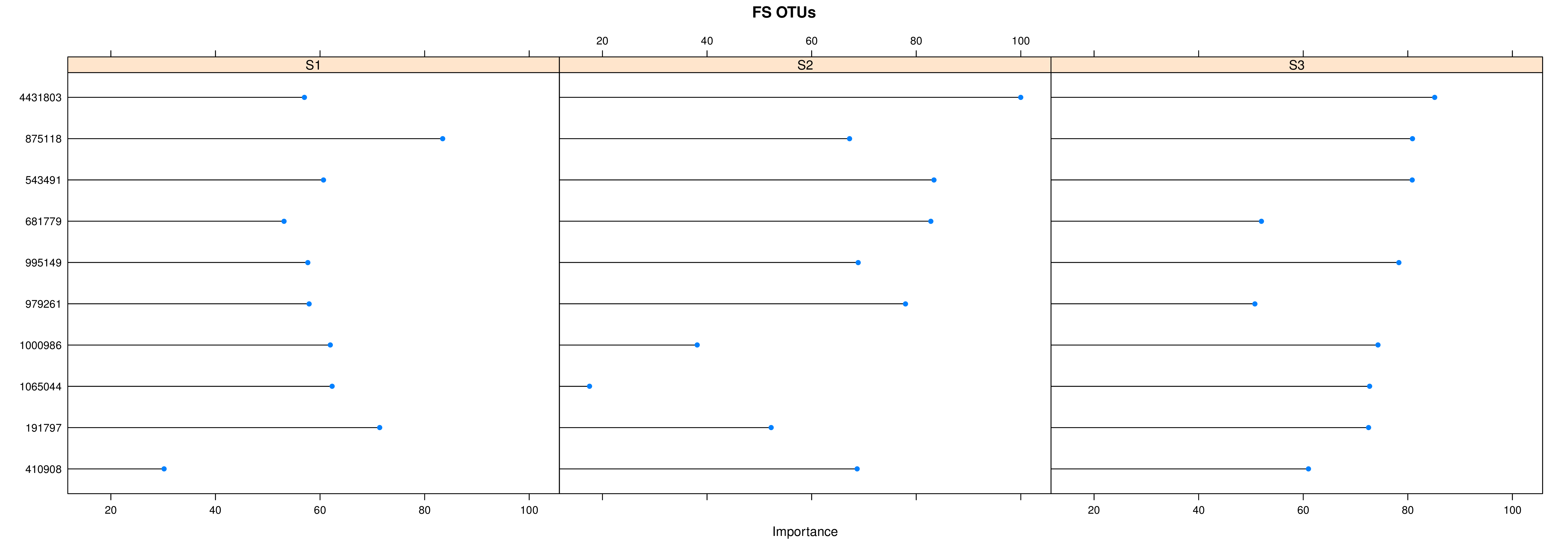


Figure S1. The feature importance of the Random Forest model for the five data sets from Knights *et al.* 2011. The key features (Green Gene IDs for OTU profile and KO/COG/Rfam IDs for PiCRUST prediction. See Table S1.) are listed on the Y axis of each plot and their scores for each categories are on X axis. The importance for each feature is calculated as explained in the paper (Liaw & Wiener, 2002) and then is scaled to 0 to 100. Features are ranked by their maximal scores across categories and only top ten features are plotted.

Table S1. The taxonomy or KO description for the IDs in Figure S2.

| 174763 | k__Bacteria; p__Firmicutes; c__Clostridia; o__Clostridiales; f__Lachnospiraceae; g__Blautia; s__ |
| --- | --- |
| 191797 | k__Bacteria; p__Firmicutes; c__Clostridia; o__Clostridiales; f__Clostridiaceae; g__; s__ |
| 222818 | k__Bacteria; p__Actinobacteria; c__Actinobacteria; o__Actinomycetales; f__Propionibacteriaceae; g__Propionibacterium; s__granulosum |
| 236684 | k__Bacteria; p__Firmicutes; c__Bacilli; o__Lactobacillales; f__Leuconostocaceae; g__Leuconostoc; s__ |
| 255732 | k__Bacteria; p__Actinobacteria; c__Actinobacteria; o__Actinomycetales; f__Micrococcaceae; g__Kocuria; s__rhizophila |
| 291846 | k__Bacteria; p__Actinobacteria; c__Actinobacteria; o__Actinomycetales; f__Corynebacteriaceae; g__Corynebacterium; s__ |
| 388506 | k__Bacteria; p__Firmicutes; c__Bacilli; o__Bacillales; f__Staphylococcaceae; g__Staphylococcus; s__ |
| 410908 | k__Bacteria; p__Actinobacteria; c__Actinobacteria; o__Actinomycetales; f__Corynebacteriaceae; g__Corynebacterium; s__ |
| 543491 | k__Bacteria; p__Actinobacteria; c__Actinobacteria; o__Actinomycetales; f__Micrococcaceae; g__; s__ |
| 681779 | k__Bacteria; p__Proteobacteria; c__Gammaproteobacteria; o__Enterobacteriales; f__Enterobacteriaceae; g__; s__ |
| 710275 | k__Bacteria; p__Proteobacteria; c__Gammaproteobacteria; o__Pseudomonadales; f__Moraxellaceae; g__Acinetobacter; s__ |
| 745556 | k__Bacteria; p__Firmicutes; c__Clostridia; o__Clostridiales; f__[Tissierellaceae]; g__Finegoldia; s__ |
| 802262 | k__Bacteria; p__Firmicutes; c__Bacilli; o__Lactobacillales; f__Streptococcaceae; g__Streptococcus; s__ |
| 811644 | k__Bacteria; p__Actinobacteria; c__Actinobacteria; o__Actinomycetales; f__Corynebacteriaceae; g__Corynebacterium; s__ |
| 875118 | k__Bacteria; p__Actinobacteria; c__Actinobacteria; o__Actinomycetales; f__Propionibacteriaceae; g__Propionibacterium; s__acnes |
| 875245 | k__Bacteria; p__Actinobacteria; c__Actinobacteria; o__Actinomycetales; f__Corynebacteriaceae; g__Corynebacterium; s__ |
| 910974 | k__Bacteria; p__Firmicutes; c__Bacilli; o__Bacillales; f__Staphylococcaceae; g__Staphylococcus; s__ |
| 979261 | k__Bacteria; p__Firmicutes; c__Bacilli; o__Bacillales; f__Staphylococcaceae; g__Staphylococcus; s__epidermidis |
| 995149 | k__Bacteria; p__Firmicutes; c__Bacilli; o__Lactobacillales; f__Aerococcaceae; g__Alloiococcus; s__ |
| 1000986 | k__Bacteria; p__Actinobacteria; c__Actinobacteria; o__Actinomycetales; f__Corynebacteriaceae; g__Corynebacterium; s__ |
| 1020410 | k__Bacteria; p__Firmicutes; c__Bacilli; o__Bacillales; f__Staphylococcaceae; g__Staphylococcus; s__ |
| 1023405 | k__Bacteria; p__Actinobacteria; c__Actinobacteria; o__Actinomycetales; f__Corynebacteriaceae; g__Corynebacterium; s__ |
| 1041758 | k__Bacteria; p__Proteobacteria; c__Gammaproteobacteria; o__Pseudomonadales; f__Moraxellaceae; g__; s__ |
| 1064036 | k__Bacteria; p__Firmicutes; c__Clostridia; o__Clostridiales; f__[Tissierellaceae]; g__Peptoniphilus; s__ |
| 1065044 | k__Bacteria; p__Actinobacteria; c__Actinobacteria; o__Actinomycetales; f__Corynebacteriaceae; g__Corynebacterium; s__ |
| 1079866 | k__Bacteria; p__Firmicutes; c__Bacilli; o__Lactobacillales; f__Streptococcaceae; g__Streptococcus; s__ |
| 1131894 | k__Bacteria; p__Cyanobacteria; c__Chloroplast; o__Streptophyta; f__; g__; s__ |
| 2101745 | k__Bacteria; p__Actinobacteria; c__Actinobacteria; o__Actinomycetales; f__Propionibacteriaceae; g__Propionibacterium; s__acnes |
| 2356875 | k__Bacteria; p__Firmicutes; c__Bacilli; o__Bacillales; f__Staphylococcaceae; g__Staphylococcus; s__ |
| 2472603 | k__Bacteria; p__Actinobacteria; c__Actinobacteria; o__Actinomycetales; f__Corynebacteriaceae; g__Corynebacterium; s__ |
| 4001495 | k__Bacteria; p__Cyanobacteria; c__Chloroplast; o__Streptophyta; f__; g__; s__ |
| 4308647 | k__Bacteria; p__Actinobacteria; c__Actinobacteria; o__Actinomycetales; f__Actinomycetaceae; g__Actinomyces; s__ |
| 4430843 | k__Bacteria; p__Bacteroidetes; c__Bacteroidia; o__Bacteroidales; f__Prevotellaceae; g__Prevotella; s__ |
| 4431803 | k__Bacteria; p__Cyanobacteria; c__Chloroplast; o__Streptophyta; f__; g__; s__ |
| 4455250 | k__Bacteria; p__Firmicutes; c__Bacilli; o__Lactobacillales; f__Streptococcaceae; g__Streptococcus; s__ |
| 4469722 | k__Bacteria; p__Bacteroidetes; c__Bacteroidia; o__Bacteroidales; f__Prevotellaceae; g__Prevotella; s__melaninogenica |
| COG0591 | Na+/proline symporter |
| COG0703 | Shikimate kinase |
| COG1252 | NADH dehydrogenase, FAD-containing subunit |
| COG1476 | Predicted transcriptional regulators |
| COG1687 | Predicted branched-chain amino acid permeases (azaleucine resistance) |
| COG1878 | Predicted metal-dependent hydrolase |
| COG2871 | Na+-transporting NADH:ubiquinone oxidoreductase, subunit NqrF |
| COG3548 | Predicted integral membrane protein |
| COG4279 | Uncharacterized conserved protein |
| COG4283 | Uncharacterized conserved protein |
| COG4325 | Predicted membrane protein |
| COG4413 | Urea transporter |
| COG4592 | ABC-type Fe2+-enterobactin transport system, periplasmic component |
| COG4603 | ABC-type uncharacterized transport system, permease component |
| COG4607 | ABC-type enterochelin transport system, periplasmic component |
| COG4710 | Predicted DNA-binding protein with an HTH domain |
| COG4913 | Uncharacterized protein conserved in bacteria |
| COG4918 | Uncharacterized protein conserved in bacteria |
| COG5406 | Nucleosome binding factor SPN, SPT16 subunit |
| COG5515 | Uncharacterized conserved small protein |
| K00001 | alcohol dehydrogenase [EC:1.1.1.1] |
| K00005 | glycerol dehydrogenase [EC:1.1.1.6] |
| K00086 | 1,3-propanediol dehydrogenase [EC:1.1.1.202] |
| K00974 | tRNA nucleotidyltransferase (CCA-adding enzyme) [EC:2.7.7.72 3.1.3.- 3.1.4.-] |
| K01455 | formamidase [EC:3.5.1.49] |
| K01547 | K+-transporting ATPase ATPase B chain [EC:3.6.3.12] |
| K01951 | GMP synthase (glutamine-hydrolysing) [EC:6.3.5.2] |
| K02071 | D-methionine transport system ATP-binding protein |
| K03308 | neurotransmitter:Na+ symporter, NSS family |
| K03667 | ATP-dependent HslUV protease ATP-binding subunit HslU |
| K05846 | osmoprotectant transport system permease protein |
| K06718 | L-2,4-diaminobutyric acid acetyltransferase [EC:2.3.1.178] |
| K07084 | NA |
| K07133 | NA |
| K09007 | GTP cyclohydrolase I [EC:[3.5.4.16](http://www.genome.jp/dbget-bin/www_bget?ec:3.5.4.16)] |
| K09686 | antibiotic transport system permease protein |
| K09790 | hypothetical protein |
| K09963 | hypothetical protein |
| RF00569 | SNORD19 |
| RF00634 | SAM-IV |
| RF00716 | mir-3 |
| RF01998 | group-II-D1D4-1 |
| RF01763 | ykkC-III |
| RF01497 | ALIL pseudoknot |
| RF00161 | Nanos 3' UTR translation control element |
| RF01666 | rox2 |
| RF00379 | ydaO/yuaA leader |
| RF01650 | C. elegans snoRNA ceN48 |
| RF00362 | Pospi_RY |


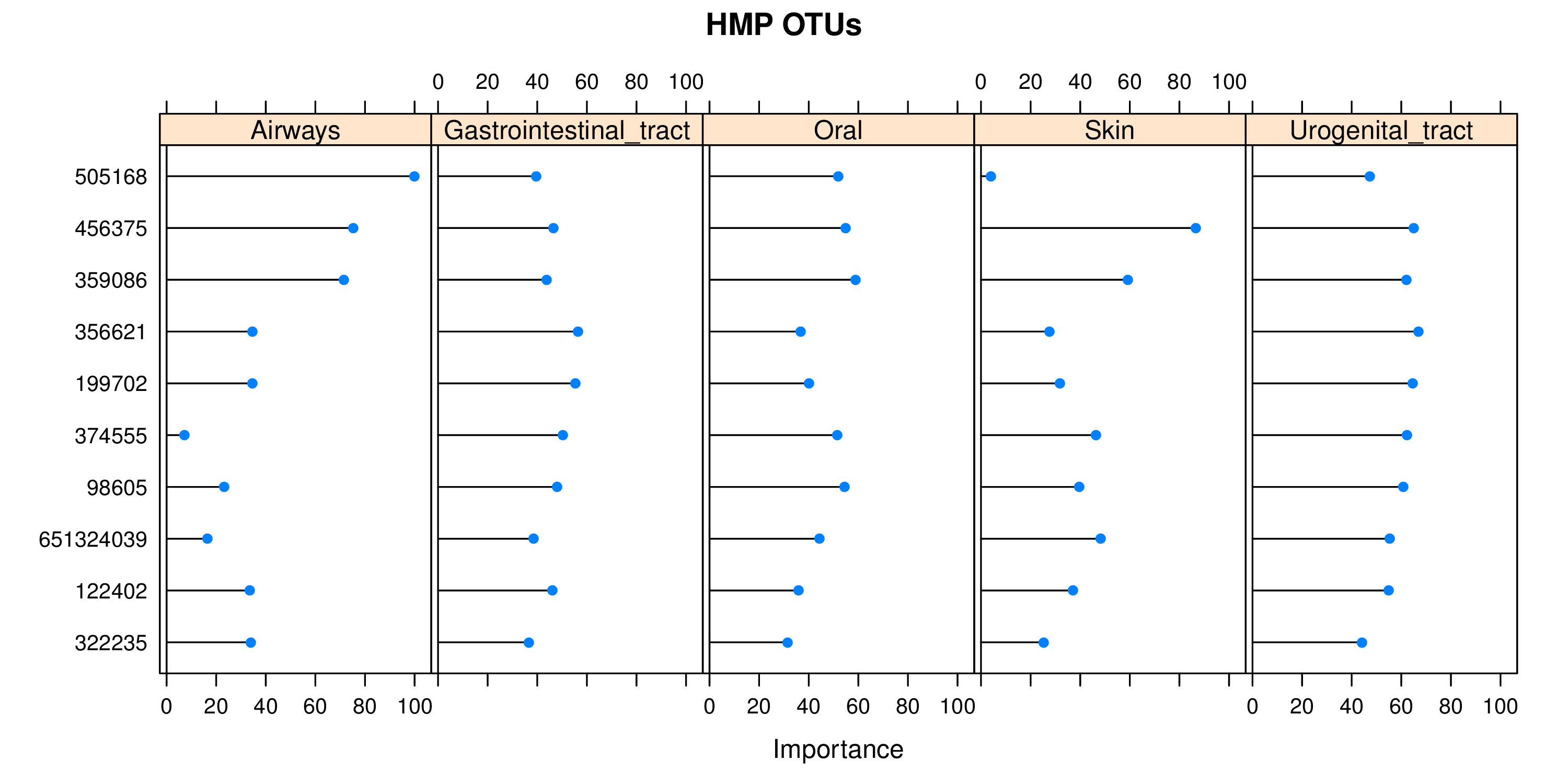


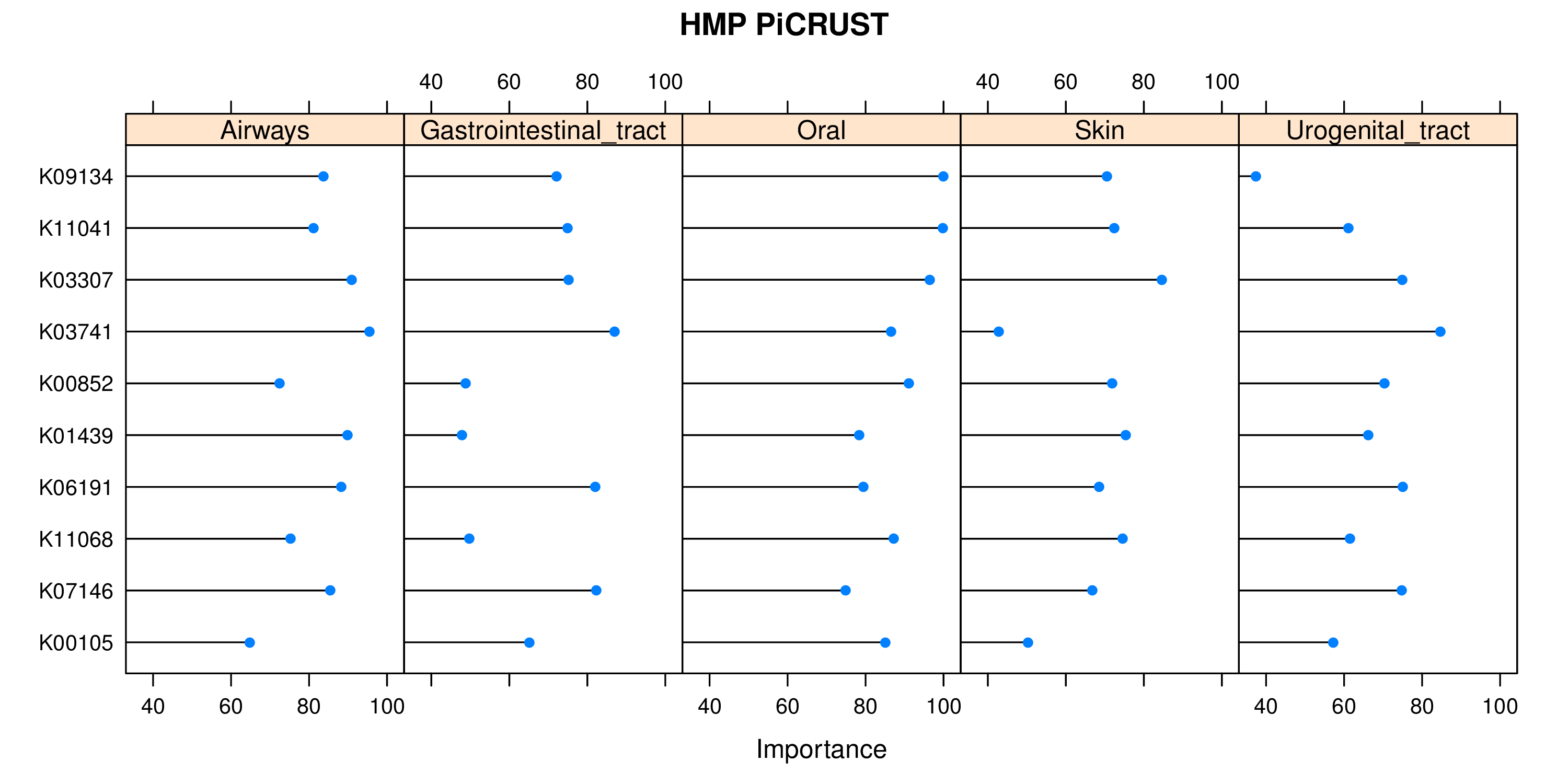

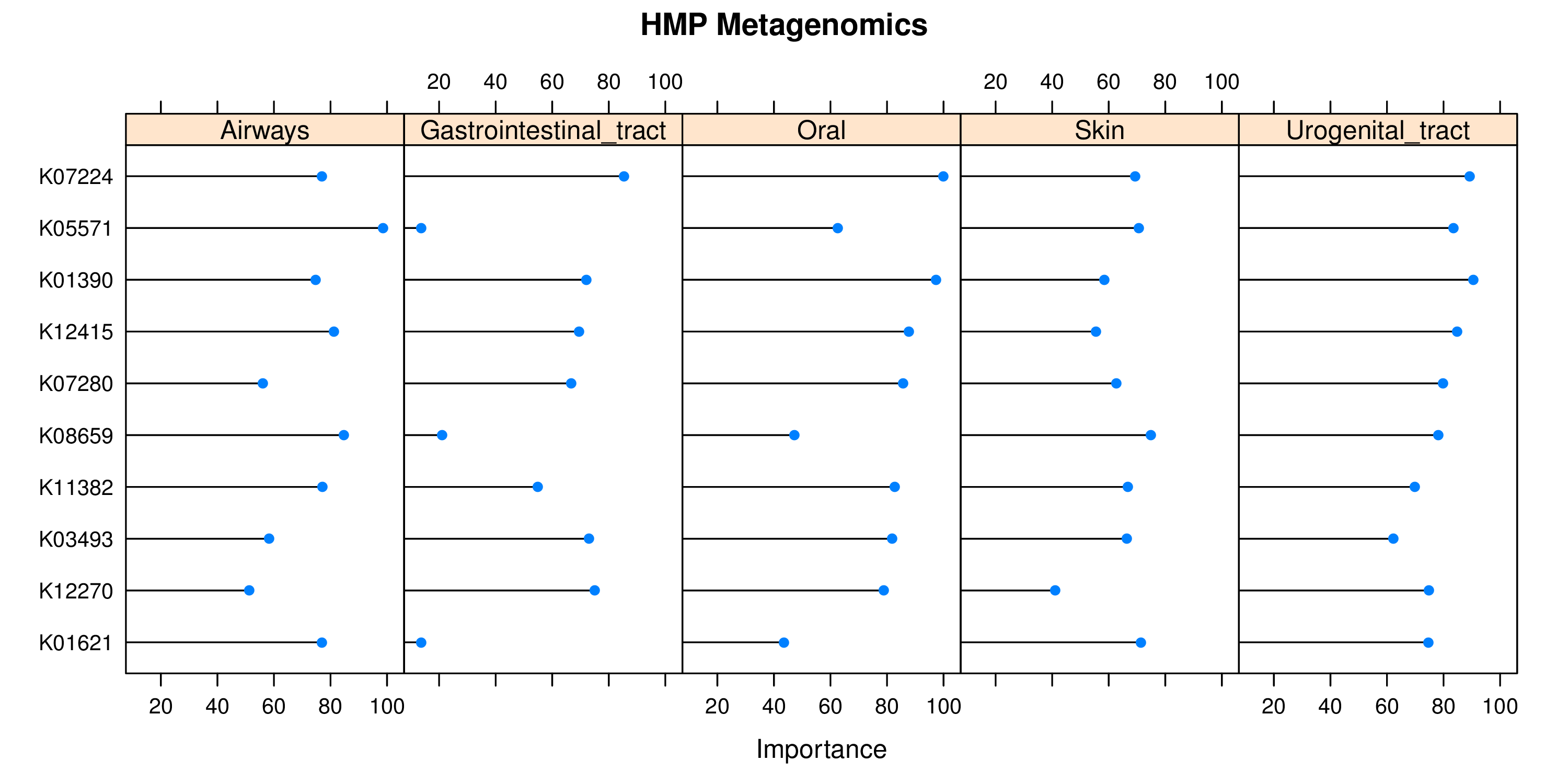
Figure S2. The feature importance of the Random Forest model for the HMP data set using the tables from PiCRUST paper. Only KO tables are available and used here. It follows the same style as Figure S1.

| 505168 | k__Bacteria; p__Actinobacteria; c__Actinobacteria; o__Actinomycetales; f__Corynebacteriaceae; g__Corynebacterium; s__ |
| --- | --- |
| 456375 | k__Bacteria; p__Actinobacteria; c__Actinobacteria; o__Actinomycetales; f__Propionibacteriaceae; g__Propionibacterium; s__acnes |
| 359086 | k__Bacteria; p__Firmicutes; c__Bacilli; o__Bacillales; f__Staphylococcaceae; g__Staphylococcus; s__ |
| 356621 | k__Bacteria; p__Bacteroidetes; c__Bacteroidia; o__Bacteroidales; f__Bacteroidaceae; g__Bacteroides; s__ |
| 199702 | k__Bacteria; p__Firmicutes; c__Clostridia; o__Clostridiales; f__Ruminococcaceae; g__Faecalibacterium; s__prausnitzii |
| 374555 | k__Bacteria; p__Firmicutes; c__Bacilli; o__Lactobacillales; f__Streptococcaceae; g__Streptococcus; s__ |
| 98605 | k__Bacteria; p__Firmicutes; c__Bacilli; o__Lactobacillales; f__Streptococcaceae; g__Streptococcus; s__ |
| 122402 | k__Bacteria; p__Bacteroidetes; c__Bacteroidia; o__Bacteroidales; f__Bacteroidaceae; g__Bacteroides; s__ |
| 322235 | k__Bacteria; p__Bacteroidetes; c__Bacteroidia; o__Bacteroidales; f__Bacteroidaceae; g__Bacteroides; s__uniformis |
| 651324039 | k__Bacteria; p__Proteobacteria; c__Gammaproteobacteria; o__Pasteurellales; f__Pasteurellaceae; g__Haemophilus; s__ |
| K09134 | hypothetical protein |
| K11041 | exfoliative toxin A/B |
| K03307 | solute:Na+ symporter, SSS family |
| K03741 | arsenate reductase [EC:1.20.4.1] |
| K00852 | ribokinase [EC:2.7.1.15] |
| K01439 | succinyl-diaminopimelate desuccinylase [EC:3.5.1.18] |
| K06191 | glutaredoxin-like protein NrdH |
| K11068 | hemolysin III |
| K07146 | UPF0176 protein |
| K00105 | alpha-glycerophosphate oxidase [EC:1.1.3.21] |
| K07224 | putative lipoprotein |
| K05571 | multicomponent Na+:H+ antiporter subunit G |
| K01390 | IgA-specific metalloendopeptidase [EC:3.4.24.13] |
| K12415 | competence-stimulating peptide |
| K07280 | hypothetical protein |
| K08659 | dipeptidase [EC:3.4.-.-] |
| K11382 | MFS transporter, OPA family, phosphoglycerate transporter protein |
| K03493 | transcriptional antiterminator |
| K12270 | accessory secretory protein Asp3 |
| K01621 | phosphoketolase [EC:4.1.2.9] |

Table S2. The taxonomy or KO description for the IDs in Figure S2.

**Reference**

Liaw A, Wiener M. (2002). Classification and Regression by randomForest*. R Ne*w**s** 2:18–22.
